# When poor sleep hurts most: procrastination as a moderator of the association between sleep quality and academic performance in medical students

**DOI:** 10.3389/fpsyg.2026.1807387

**Published:** 2026-03-25

**Authors:** Michael Nazmifar, Rachel Lloyd, Lauren Walkon, Tina Izad, Changiz Mohiyeddini

**Affiliations:** Oakland University William Beaumont School of Medicine, Rochester, MI, United States

**Keywords:** academic performance, medical students, moderation analysis, procrastination, sleep quality

## Abstract

The demanding nature of medical training elevates the risk of sleep disruption and may compromise academic performance. At the same time, procrastination is a prevalent self-regulatory difficulty among students that has been independently linked to poorer academic outcomes. Although both sleep quality and procrastination are two critical self-regulatory variables that have been linked to performance, their interplay is not well understood. The present cross-sectional study examined whether procrastination moderates the association between sleep quality and academic performance in medical students. An *a priori* power analysis indicated that a minimum of 119 participants was required. The final sample comprised 125 medical students (M age = 25.6 years, 54% female). Participants completed self-report measures of sleep quality (Pittsburgh Sleep Quality Index; PSQI), procrastination (Behavioral and Emotional Academic Procrastination Scale; BEPS), and academic performance (composite exam grades). Hierarchical regression analyses showed that poorer sleep quality and greater procrastination were each significantly associated with lower academic performance. In addition, the interaction between sleep quality and procrastination was significant, accounting for an additional 2.7% of variance in performance. The simple slope analysis supported the main hypothesis: the negative association between poor sleep and performance was stronger at higher levels of procrastination, whereas the association was weaker among students with lower procrastination. Though the additional variance was modest (Δ*R*^2^ = 0.027), results may inform evidence-based strategies to support student well-being and success.

## Introduction

Medical education is a highly demanding and challenging experience. Medical students face enormous workloads, long hours, and many assignments, exams, and hands-on training that must be mastered in a highly competitive (Ensz and Mohiyeddini, 2023).

The decline in sleep quality is one of the most pervasive, yet often underappreciated, consequences of medical education. Surveys of medical students in the US regularly indicate elevated rates of insufficient sleep, irregular sleep–wake patterns, and poor subjective sleep quality (Brick et al., 2020). This pervasive pattern of sleep deprivation and poor sleep quality has several severe repercussions for learning and academic performance of medical students. That is due to the well-established fact that the cognitive functions essential for learning (e.g., memory consolidation, attention, and information processing) are highly dependent on the quality of sleep (Hudson, 2023). Hence, sleep deprivation and poor sleep quality undermine the very cognitive processes medical students rely on to perform and succeed in their demanding curriculum, leading to decreased academic achievement and poorer examination performances.

Independent of sleep quality, procrastination—the voluntary and unnecessary delay of intended tasks despite anticipating negative consequences—represents a stable, trait-like individual difference rooted in self-regulatory failure (Sirois et al., 2022; Steel, 2007). Meta-analytic evidence demonstrates that procrastination is strongly and consistently predicted by low conscientiousness and its facets, including self-control, distractibility, organization, and achievement motivation (Steel, 2007). Longitudinal research confirms that procrastination exhibits high temporal stability across time and situations (Steel and Klingsieck, 2016), and is best understood as a persistent behavioral disposition rather than a transient response to situational factors such as sleep loss. Among medical students specifically, trait procrastination has been linked to impaired cognitive capacity and poorer academic outcomes through compromised self-regulatory processes (Pikó et al., 2023).

Critically, although both poor sleep quality and procrastination reflect deficits in self-regulation (Klingsieck, 2013), they impair performance through different mechanisms: poor sleep depletes cognitive resources essential for learning—memory consolidation, attention, and executive function (Hudson, 2023)—whereas procrastination reflects a failure to deploy behavioral strategies such as planning and timely task initiation (Steel and Klingsieck, 2016).

The theoretical basis for a moderation framework rests less on a state–trait distinction per se than on the proposition that sleep quality and procrastination impair performance through different mechanisms. Students who lack these compensatory behavioral strategies may be disproportionately affected when cognitive resources are also depleted by poor sleep, because neither pathway is functioning adequately. Extending this moderation logic to the academic domain, we propose that the degree to which poor sleep quality undermines academic performance depends on a student’s standing on trait procrastination. Students high in procrastination lack the compensatory self-regulatory strategies—such as effective time management, task prioritization, and sustained effort—that might otherwise buffer the cognitive costs of poor sleep (Steel and Klingsieck, 2016). In contrast, students low in procrastination may compensate for the cognitive deficits associated with poor sleep through established study habits, strategic planning, and disciplined task engagement. In this view, procrastination functions as a vulnerability factor that amplifies the negative association between poor sleep quality and academic performance.

Given this theoretical rationale, the present study examines whether trait procrastination moderates the association between self-reported sleep quality and academic performance among medical students. Specifically, we test whether the negative relationship between poor sleep quality and academic performance is stronger among students with higher levels of procrastination.

However, to our knowledge, no study has explored procrastination as a moderator of the relationship between sleep quality and academic performance. This study addresses this gap among medical students, with implications for targeted interventions addressing sleep hygiene and self-regulation during medical training.

We hypothesize:

*H1*: Self-reported poor sleep quality will be negatively associated with academic performance as measured by examination outcomes.

*H2*: Procrastination will be negatively associated with academic performance.

*H3* (Moderation): Procrastination will moderate the association between self-reported sleep quality and academic performance, such that the negative association between sleep quality and academic performance will be more pronounced among students with higher levels of procrastination.

## Methods

### Power analysis

An *a priori* power analysis (G*Power 3.1; Faul et al., 2009) for detecting a significant Δ*R*^2^ with three predictors (*α* = 0.05, power = 0.80, *f*^2^ = 0.04) indicated a minimum sample of 119 participants. Our sample of *N* = 125 meets this threshold.

### Sample and procedure

Medical students at Oakland University William Beaumont School of Medicine were invited to participate in this study between February and May 2025 via university email. Students were provided with a link to the online survey, and informed consent was obtained.

137 students responded to the survey. Twelve subjects were excluded who started but did not finish the survey. The final sample consisted of 125 medical students (54% self-identified as female and 46% as male). The average age of participants was *M* = 25.57 years (SD = 1.81).

### Ethical considerations

The study was approved by the Oakland University Institutional Review Board (September 2024). Informed consent was obtained online; participants could skip questions or withdraw at any time without penalty.

## Measurements

### Sleep quality

Sleep quality was assessed using the Pittsburgh Sleep Quality Index (PSQI). PSQI is widely used and is acknowledged as a valid and reliable approach for evaluating subjective sleep quality (Buysse et al., 1989). The PSQI consists of 19 self-report items assessing seven components (plus 5 items rated by a bed partner, which are not used in scoring): subjective sleep quality, sleep latency, sleep duration, habitual sleep efficiency, sleep disturbances, use of sleep medication, and daytime dysfunction. Each component is scored on a scale from 0 to 3, with the sum yielding a global score ranging from 0 to 21. Higher scores reflect poorer sleep quality. Because the PSQI assesses habitual sleep patterns over the preceding month, scores reflect stable between-person differences rather than short-term fluctuations.

The PSQI has demonstrated strong reliability and validity across diverse populations and has been recognized as a reliable tool for both clinical and research settings worldwide. The internal consistency of the PSQI global score, calculated across its seven component scores, was adequate (Cronbach’s *α* = 0.79).

### Academic performance

Academic performance was assessed using a composite score derived from coded course grades. This method is commonly used in medical education research where GPA is unavailable due to competency-based or pass/fail grading policies (Bloodgood et al., 2009). This standardized approach ensures consistency across students completing the same curriculum. To ensure comparability across the sample, we used exam scores from six required first-year medical school courses that were identical for all cohorts. These standardized courses are completed by all students, providing a uniform academic metric. In contrast, later-year coursework involves greater variability due to elective choices and clinical rotations, making cross-student comparisons less valid.

The six courses included: Anatomical Foundations of Clinical Practice 1, Anatomical Foundations of Clinical Practice 2, Biomedical Foundations of Clinical Practice 1, Biomedical Foundations of Clinical Practice 2, Hematology and Oncology, and Neurology.

A composite academic performance score was calculated for each participant by averaging the coded grades across the six courses. This resulted in a continuous score ranging from Zero (extremely low academic performance) to 100 (very high academic performance), providing a reliable and standardized measure of academic achievement.

### Academic procrastination

Academic procrastination was measured using the Behavioral and Emotional Academic Procrastination Scale (BEPS; Bobe et al., 2024). BEPS is a 6-item self-report scale that measures academic procrastination across two subscales: Delay (3 items capturing voluntary postponement of study tasks) and Subjective Discomfort (3 items capturing feelings of guilt, worry, and distress during the delay). Items are rated on a 5-point scale from 1 (never) to 5 (always). The BEPS has demonstrated strong reliability and validity across multiple empirical studies (Bobe et al., 2024; Grunschel et al., 2024); in addition to its two subscales, an overall score can be computed to capture the general tendency toward academic procrastination, which was used in the present study, yielding excellent internal consistency (Cronbach’s *α* = 0.91).

### Analytic approach

Because the study is cross-sectional, these hypotheses are tested as statistical associations; the moderation hypothesis tests whether the magnitude of the sleep–performance association differs across procrastination levels.

Prior to the statistical analyses, the database was checked for potential encoding errors and aberrant values. All calculations were performed using SPSS v.30 (SPSS Inc., Chicago, IL). Data are presented as mean ± SD. Cases with missing values were excluded list-wise. The distribution of variables was examined using the Kolmogorov–Smirnov test. Standard diagnostic checks were conducted to evaluate regression assumptions: residual plots were inspected for linearity and homoscedasticity, and variance inflation factors (VIFs) were computed to assess multicollinearity. No violations of assumptions were detected, and all VIFs were below 2.0, indicating low multicollinearity.

Pearson’s correlation coefficients (two-tailed) were calculated to examine the associations among sleep quality (PSQI total score), procrastination, and academic performance.

All variables were mean-centered prior to computing interaction terms to ensure comparability across measures (Dunlap and Kemery, 1987).

Age, gender, and year in medical school were considered as potential covariates. Preliminary analyses indicated that these variables were not significantly correlated with sleep quality, procrastination, or academic performance (|r| ≤ 0.10, ps ≥ 0.24). Consistent with recommendations to avoid unnecessary statistical control when covariates lack empirical or theoretical justification (Becker, 2005; Bernerth and Aguinis, 2016), these variables were not included in the primary analyses.

To evaluate the robustness of the findings, all regression models were re-estimated including age, gender, and year in medical school as covariates. The pattern of results—including the significance, direction, and magnitude of the main effects and the sleep quality × procrastination interaction—remained unchanged. Accordingly, results from the more parsimonious models are reported.

To test for moderation, hierarchical multiple regression analyses were conducted. In the first step, PSQI scores and procrastination were entered as predictors of academic performance. In the second step, the interaction term (PSQI × procrastination, computed after centering the predictors) was added to the model. Statistical significance was set at *p* < 0.05 (two-tailed). A significant interaction effect was followed up by simple slopes analysis to probe the nature of the moderation.

### Common method bias assessment

Because all variables were self-reported, Harman’s single-factor test was conducted to assess common method variance (Podsakoff et al., 2003). The first unrotated factor accounted for 41% of variance, below the conventional 50% threshold.

## Results

### Descriptive statistics

Table 1 displays descriptive statistics and bivariate correlations among the study variables.

**Table 1:** Means, standard deviations, and correlations among study variables (*N* = 125).

Variable	*M*	SD	1	2	3
1. Sleep quality (PSQI)	9.2	2.40	—		
2. Procrastination	3.2	1.61	0.37**	—	
3. Academic performance	84.82	11.7	−0.44**	−0.57**	—

Higher PSQI scores = poorer sleep quality. **p < 0.001.

For the main variables, Pittsburgh Sleep Quality Index (PSQI; 0–21, higher scores indicate poorer sleep quality) had a mean of 9.20 (SD = 2.40; *n* = 125). Procrastination (range 1–5) averaged 3.20 (SD = 1.61; *n* = 125). Academic performance ranged from ~61 to ~99 with a mean of 84.82 (SD = 11.70; *n* = 125). After centering, the means of PSQI and Procrastination were 0 by design; the product term (PSQI× Procrastination) had a mean of *M* = 4.42 (SD = 11.65).

### Zero-order correlations

Poorer sleep quality (higher PSQI) was negatively associated with academic performance, *r* = −0.44, *p* < 0.001. Higher procrastination was also negatively associated with performance, *r* = −0.57, *p* < 0.001. PSQI and procrastination were positively associated, *r* = 0.37, *p* < 0.001.

### Moderation analysis (hierarchical regression)

Model diagnostics: Collinearity diagnostics showed small condition indices (≤ ~ 1.50) across steps, suggesting no multicollinearity concerns among PSQI, Procrastination, and their interaction.

To test whether procrastination moderates the association between sleep quality and academic performance, a two-step hierarchical linear regression was conducted with academic performance as the outcome. Centered main effects of PSQI and procrastination were entered in Step 1, accounting for 38.0% of variance in performance, *F* (2,122) = 37.43, *p* < 0.001. In Step 2, adding the interaction term explained an additional 2.7% of variance, Δ*R*^2^ = 0.027, ΔF (1,121) = 5.52, *p* = 0.020. Both main effects remained significant (PSQI: *B* = −0.427, *p* < 0.001; Procrastination: *B* = −3.407, *p* < 0.001), and the interaction was significant (*B* = −0.165, SE = 0.070, *t* = −2.35, *p* = 0.020), indicating that procrastination moderated the association between sleep quality and academic performance.

To rule out the possibility that the significant interaction reflected a curvilinear (nonlinear) relationship between the predictors and academic performance rather than a true moderation effect, supplementary analyses were conducted in which quadratic terms for PSQI and procrastination were entered alongside the linear main effects before adding the interaction term. Neither the quadratic term for PSQI nor the quadratic term for procrastination was significant, and the PSQI × Procrastination interaction remained significant after their inclusion. These results indicate that the observed moderation effect is not attributable to nonlinear main effects of either predictor.

### Nature of the moderation

**Figure 1:**
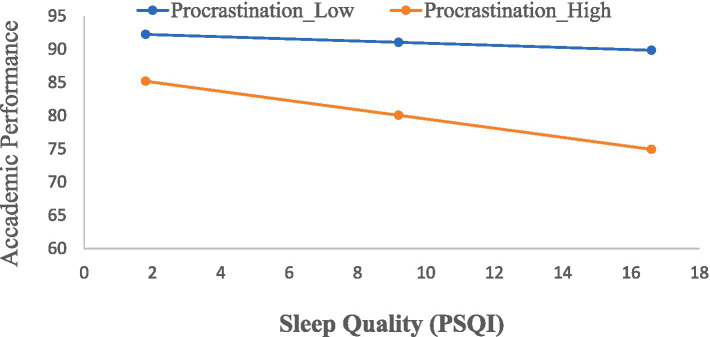
Interaction between sleep quality (PSQI) and procrastination in relation to academic performance (exam composite scores). Predicted values are shown for students reporting low (−1 SD) and high (+1 SD) levels of procrastination. Higher PSQI scores indicate poorer sleep quality. As illustrated, the negative association between poor sleep quality and academic performance is stronger among students with higher procrastination, whereas this association is weaker among students with lower procrastination.

Simple-slope computations (Figure 1) based on the centered coefficients indicate that the negative association between poorer sleep quality and academic performance is stronger at higher levels of procrastination. At high levels of procrastination (+1 SD), the simple slope of PSQI on performance was significant [*B* = −0.69, SE = 0.15, *t* = −4.60, *p* < 0.001, 95% CI (−0.99, −0.39)], indicating that poorer sleep quality was strongly associated with lower performance among high procrastinators. At low levels of procrastination (−1 SD), the simple slope was not significant [*B* = −0.16, SE = 0.14, *t* = −1.14, *p* = 0.256, 95% CI (−0.44, 0.12)], indicating that sleep quality was essentially unrelated to performance among low procrastinators. These results are consistent with a moderation pattern in which the sleep–performance association varies by procrastination level in this sample.

## Discussion

The present study examined whether procrastination moderates the association between sleep quality and academic performance among medical students. Consistent with prior research, poorer sleep quality (Brick et al., 2020; Okano et al., 2019) and greater procrastination (Kim and Seo, 2015) were each significantly associated with worse exam performance. Beyond these independent associations, the results provided cross-sectional evidence for a significant interaction: the link between sleep quality and performance varied depending on levels of procrastination. Importantly, the moderation effect remained statistically significant when controlling for age, gender, and year in medical school, indicating that the observed interaction is not attributable to basic sociodemographic differences.

Although sleep quality and procrastination were moderately correlated (*r* = 0.37), this association does not imply construct redundancy. The BEPS (Bobe et al., 2024) captures the voluntary delay of academic tasks accompanied by subjective discomfort—a dispositional tendency that is conceptually and empirically distinct from bedtime procrastination, which refers specifically to the failure to go to bed at an intended time despite no external obstacles (Kroese et al., 2014). The procrastination scores in the present study therefore reflect habitual academic self-regulatory failure rather than a disposition to delay sleep onset per se. This distinction supports the interpretation that the observed moderation captures the convergence of two functionally different self-regulatory vulnerabilities—depleted cognitive resources due to poor sleep, and deficient behavioral strategies for academic task management—rather than a statistical artifact of overlapping constructs.

Both sleep quality and procrastination were measured as trait-like constructs; however, the PSQI’s one-month window may conflate stable patterns with temporary fluctuations. Our moderation model therefore tests how characteristic sleep quality and dispositional procrastination jointly relate to performance. Future studies using sleep diaries or actigraphy could disentangle state and trait components of this interaction. Notably, among low procrastinators, sleep quality was not significantly associated with performance, suggesting that effective self-regulatory habits may buffer the academic consequences of poor sleep, whereas students lacking these strategies are more vulnerable to sleep-related performance decrements.

The modest increment in explained variance (Δ*R*^2^ = 0.027) is consistent with the typically small moderation effects observed in observational psychological research (Aguinis et al., 2005) and should not be interpreted in isolation as evidence against practical relevance. The simple-slope results illustrate the substantive meaning of this interaction more clearly: among students with high procrastination (+1 SD), each unit increase in PSQI score was associated with a 0.69-point decrease in exam performance (*B* = −0.69, *p* < 0.001), whereas among students with low procrastination (−1 SD), the same unit increase in PSQI was associated with only a 0.16-point decrease that did not reach statistical significance (*B* = −0.16, *p* = 0.256). This more than fourfold difference in the magnitude of the sleep–performance gradient across procrastination levels suggests that the interaction, while modest at the group level, may carry meaningful implications for identifying which students are most academically vulnerable to poor sleep. In a high-stakes educational environment such as medical training, even modest performance differences can carry significant consequences for academic progression and licensing outcomes.

These findings extend previous research by considering the combined role of two self-regulatory factors that have generally been studied independently. Whereas prior work has shown that sleep quality is associated with cognitive performance and learning outcomes, and that procrastination is associated with time-management difficulties and lower academic achievement (Steel and Klingsieck, 2016), the present findings add cross-sectional evidence that these two factors interact—though longitudinal studies are needed to establish the direction and temporal dynamics of these relationships.

From a practical standpoint, should these associations be confirmed longitudinally, the findings suggest that interventions addressing procrastination may be particularly relevant for students who also report poor sleep, and that sleep hygiene programs may be especially beneficial for students with pronounced procrastination tendencies. Given the cross-sectional nature of the present data, such implications remain preliminary and warrant empirical testing.

More broadly, these findings align with structural equation modeling evidence showing that multiple interacting lifestyle and behavioral variables—including sleep and self-regulatory behaviors — jointly shape health-related quality of life and academic functioning in university student populations (Sánchez-Ojeda et al., 2024), underscoring the value of holistic, multi-component approaches to student well-being.

Overall, these findings emphasize the need for holistic approaches to student well-being that address both sleep and self-regulatory skills in medical education.

## Limitations

There are several limitations to the present study. First, the cross-sectional design precludes causal inference and cannot resolve the directionality of the observed moderation. Although we framed procrastination as the moderator based on trait-like stability, the interaction is mathematically symmetric. It is possible that poor sleep quality exacerbates the negative effects of procrastination, or that a third variable influences both. Longitudinal or experimental studies that assess sleep and procrastination at multiple time points are needed to clarify the temporal and potentially reciprocal nature of these relationships.

Second, although the *a priori* power analysis indicated adequate power for detecting the targeted effect size, the study sample remains relatively small (*N* = 125). In addition, participants were drawn from a single medical school in the United States, which limits generalizability to other student populations, institutions, educational systems, and cultural contexts. Medical education varies substantially across countries in terms of curriculum structure, grading practices, student demographics, and workload intensity. Future studies should seek to replicate the present moderation pattern in larger, multi-institution samples and in medical schools operating under different educational models—for example, graduate-entry versus undergraduate-entry programs—as well as in non-Western contexts.

Third, the exclusive reliance on self-report measures remains a limitation (Podsakoff et al., 2003). As a precautionary step, Harman’s single-factor test was conducted; the first unrotated factor accounted for 41% of the variance, below the conventional 50% threshold, providing partial reassurance that common method variance is unlikely to account entirely for the observed effects. Nonetheless, self-reported sleep quality may not accurately reflect objective sleep parameters, and self-reported grades may differ from institutional records. Future research should incorporate objective measures—such as actigraphy or polysomnography for sleep and verified academic records for performance—alongside behavioral tracking of study engagement, to provide more rigorous tests of the moderation model.

Fourth, the assessment of academic performance was based on examination grades only, which, while important, may not fully capture the wide range of competencies required in medical education (e.g., clinical reasoning, hands-on skills, or interpersonal competencies).

Fifth, the moderation model focused exclusively on procrastination as a potential moderator. Other psychological and contextual factors, such as stress, coping strategies, social support, or workload intensity, may also moderate the relationship between sleep quality and academic outcomes but were not considered here. Finally, although the interaction effect was statistically significant, the additional variance explained was modest, indicating that further research is needed to identify additional factors contributing to medical students’ academic performance.

Despite these limitations, the present study offers novel insights into how procrastination may condition the association between sleep quality and academic performance, providing a foundation for future longitudinal and multi-method investigations.

## Data Availability

The raw data supporting the conclusions of this article will be made available by the authors, without undue reservation.
